# The seismic signature of lunar ice

**DOI:** 10.1126/sciadv.adz7220

**Published:** 2026-07-31

**Authors:** Harrison P. Lisabeth, Nicholas Schmerr, Matthew Siegler

**Affiliations:** ^1^ Lawrence Berkeley National Laboratory, 1 Cyclotron Rd, Berkeley, CA 94720, USA.; ^2^ University of Maryland, College Park, 8000 Regents Dr. College Park, MD 20742, USA.; ^3^ University of Hawaii, 2444 Dole St, Honolulu, HI 96822, USA.

## Abstract

Identifying water ice deposits on the Moon is critical for enabling long-term scientific investigations and supporting future lunar infrastructure. Although multiple lines of evidence suggest that substantial quantities of poleward water exist within the lunar near-surface, the precise location, abundance, and distribution of these ice resources remain largely unconstrained. The presence of ice in the regolith alters geophysical properties that can be quantitatively characterized using geophysical techniques analogous to those employed in terrestrial subsurface resource exploration. In this study, we integrate experimental measurements of icy regolith simulants obtained through synchrotron x-ray microtomography with advanced rock physics modeling and a thermal model of the lunar near-surface. This multi-pronged approach enables us to construct realistic seismic models for theoretical ice deposits at the lunar south pole. Our methodology allows us to link variations in seismic properties to the presence and distribution of ice, providing a framework for using seismic methods to infer both the location and the abundance of subsurface ice deposits. Our results underscore that seismic exploration techniques are a robust tool for characterizing lunar water resources. The insights gained from these models not only enhance our understanding of the physical state of the lunar regolith but also pave the way for optimizing geophysical survey strategies in upcoming lunar missions.

## Teaser

Near surface geophysics, a powerful imaging tool on Earth, is an effective method for identifying water resources on the Moon.

## INTRODUCTION

Over the next century, space exploration will realize the possibility of long-term human outposts and infrastructure on the surfaces of the Moon and Mars. Unlike Earth, the key in-situ energy resources on extraterrestrial bodies are not hydrocarbons, they are hydrogen, oxygen, water and other volatile compounds. There has been evidence for trace amounts of water trapped in lunar minerals for more than a decade (*1*), but more recent studies have identified signs that there may be free water stored as ice within the regolith (*2*). Evidence for water in the lunar regolith from remote sensing shows that the lunar south pole in particular has a substantial water resource (*3*). Recent spectroscopic data (*4*) suggests that there is between 100 and 400 mg/g of water in the regolith, far exceeding previous assumptions. While remote sensing approaches establish the presence of ice, they only poorly constrain its abundance at depth, and the challenge will be to identify deposits that are reasonably minable for industrial use. Planned robotic and crewed missions to the moon in the next decade will provide opportunities to apply the tools of terrestrial near surface geophysics to locating crucial lunar resources.

The preliminary work that has been done to understand the physical properties of regolith does suggest some possible targets for seismic imaging of volatiles in the lunar subsurface. While most studies focus on the geotechnical behavior of lunar soil (*5*, *6*) and planetary scale seismology (*7*, *8*), the few studies that do address fundamental geophysical properties of lunar regolith relevant to near surface imaging suggest unique behavior not commonly observed in terrestrial materials; for instance, anomalously low seismic attenuation (*9*, *10*), a phenomenon attributed to notable electrostatic adhesion between particles in the dry lunar environment (*11*), which indicates that volatiles play a crucial role in controlling the acoustic behavior of lunar regolith (*12*), since adsorbed volatiles dissipate these forces (*13*). The mechanical properties of lunar soil are also known to be highly inelastic (*6*) and pressure dependent (*14*). These observations suggest that the presence of volatiles within regolith may have a strong geophysical signature, especially visible in measurements of seismic properties. To our knowledge, the explicit seismic consequences of macroscopic ice distributions within the shallow subsurface have never been modeled. Here we integrate microstructural observations, ice-stability estimates, effective-medium elasticity, and full 3-D wavefield simulations to quantify how ice alters seismic velocity, diffraction, and scattering in realistic lunar conditions. By providing these quantitative predictions, our work offers a concrete set of diagnostic features that future robotic and crewed seismic experiments can use to identify and map volatile-rich horizons, filling a critical gap between remote-sensing evidence for polar ice and the geophysical measurements that will ultimately be needed to assess its accessibility.

## RESULTS

### Modeling the effect of ice on seismic velocities

Determining the most appropriate model for the ice content-dependence of seismic velocity requires an assessment of the microstructure of the ice-regolith aggregates. To estimate the effective elastic behavior of a complex aggregate from the elastic properties of constituent elements, effective medium models can be employed. The Voigt, Reuss, and Hashin-Shtrikman (*15*) models are all examples of effective medium models used in rock physics to estimate the overall mechanical properties of composite materials with different microstructures. Each of these models is applied based on the characteristics of the microstructure of icy regolith.

The effective elastic behavior of the ice-bearing regolith is approximated using bounding models that define the isostrain and isostress limits of composite media. The Voigt model represents the upper (isostrain) bound where all phases experience equal strain. This model may be approximately appropriate if ice is sparse and grain packs are largely grain-supported. The Reuss model represents the lower (isostress) bound where all phases experience equal stress. This may be appropriate if ice deposits are abundant or focused in lenses where grains are supported in an icy matrix. Natural granular materials such as lunar regolith typically fall between these bounds. These models can be joined into a Voigt-Reuss-Hill (VRH) model, which averages the two. The VRH model is often used when the microstructure of the composite is neither perfectly continuous nor entirely discontinuous, and a reasonable estimate of the overall properties is required.

If a material meets certain assumptions, the Hashin–Shtrikman (HS) bounds define the tightest theoretical limits on effective elastic moduli for isotropic, two-phase composites, depending only on the constituent moduli and volume fractions. They are commonly used to estimate upper and lower bounds on the effective properties (such as stiffness, conductivity, or other mechanical and physical properties) of a composite material with an isotropic microstructure. These bounds help provide a range within which the actual effective properties of the composite are expected to fall. The actual effective property of the composite material should fall within the range defined by these upper and lower bounds. Averaging the upper and lower bounds often provides an accurate estimate of true variations in effective properties. Mathematical details of these models can be found in the Supplementary Materials. The choice of the most appropriate effective medium model depends on the nature of the microstructure in the composite material you are studying.

### Estimating the elastic moduli

Shear modulus (μ) and bulk modulus (K) of a material can be calculated from its density (ρ) and the velocities of acoustic waves that propagate through the material (*16*). Both shear waves (S-waves) and compressional waves (P-waves) are used for the calculations. Shear modulus, also known as the modulus of rigidity, measures a material’s resistance to shearing or deformation by shear stress. Bulk modulus measures a material’s resistance to volume changes under hydrostatic pressure. The relationship between these parameters can be expressed in terms of the material’s density and the wave velocities as follows

$$\mu =\rho V_{S}^{2}$$ (1)

$$K=\rho \left(V_{P}^{2}-\frac{4}{3}V_{S}^{2}\right)$$ (2)

The procedure is straightforward to calculate the moduli of a regolith with completely ice-filled pore space. We measure the elastic velocities in icy regolith analogs using ultrasonic sensors and use these values directly to parameterize our model (details in Supplementary Material). We can directly take the density of our sample, 2.103 g/cm^3^, and the shear and compressional velocities we have measured at the completely frozen condition as our V_P_ and V_S_, and recover a shear modulus of 7.16 GPa and a bulk modulus of 22.19 GPa.

**Fig. 1.**
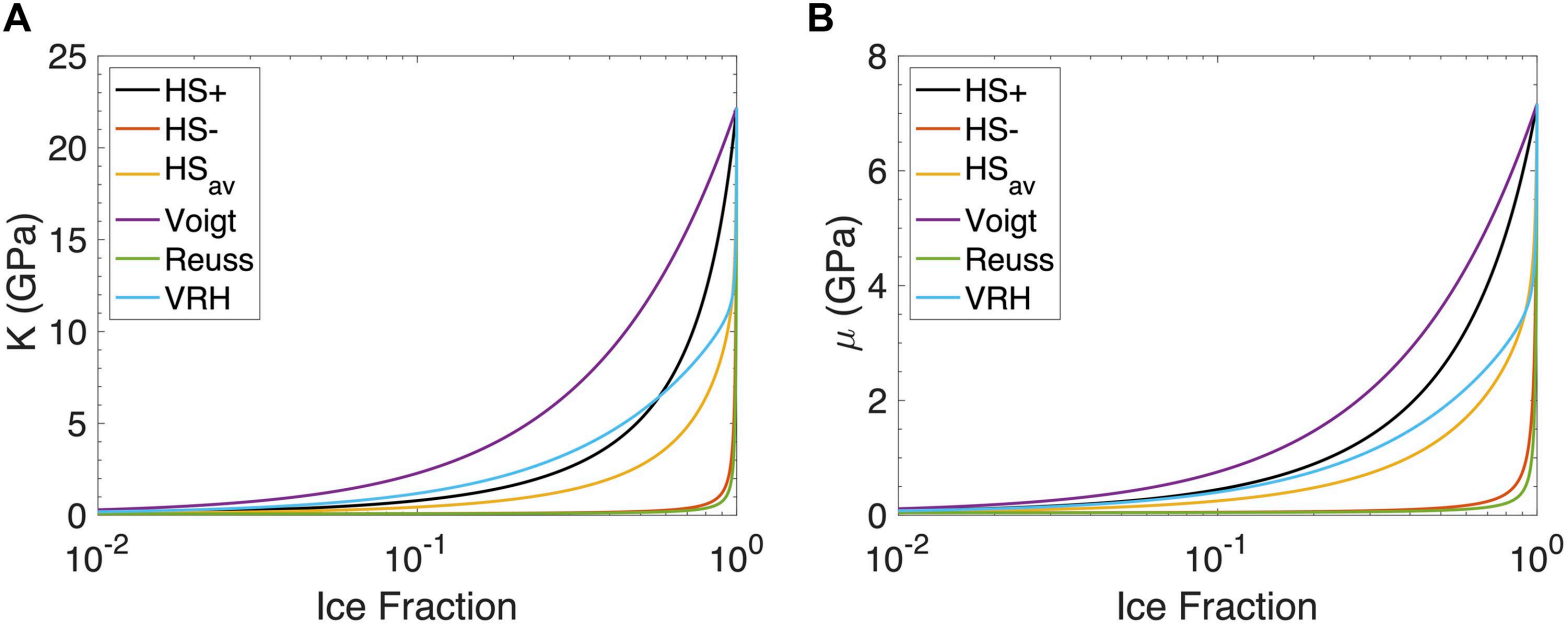
Effective medium models for icy regolith. Elastic moduli as a function of ice fraction considering the Reuss, Voigt, and V-R-H, and H-S models of ice-infused regolith material properties. (**A**) Bulk and (**B**) shear modulus.

For the ice-free reference, we use ultrasonic velocities from the Apollo 17 rock-powder dataset (*17*) obtained under 1 bar hydrostatic conditions, which most closely approximate our nominally dry laboratory environment. These samples are compositionally similar (basaltic glass ± plagioclase) to the JSC-1A simulant used here. Although mineralogical differences exist, using published values avoids desiccation artifacts that arise when oven-drying fine powders in air. Using these values and a dry regolith sample density of 1.510 g/cm3, we recover an ice-free shear modulus of 0.84 GPa and bulk modulus of 4.41 GPa. Using our estimated moduli as endmembers, the results of our rock physics models are presented in Fig. 1, A and B. This approach recovers very similar values to those modeled using a purely digital approach at low ice fractions (*18*). The various models result in a spread of predicted moduli, so it is clear that the proper assessment of the microstructure and selection of model is essential for a reliable result.

### Measurement of icy microstructure

To characterize a realistic icy microstructure in regolith, we performed in-situ x-ray microtomography of our experimental materials at the Advanced Light Source. Measurements were made on a regolith simulant from a cinder cone in Arizona, commonly referred to at JSC-1A (*19*). The samples are coarse basaltic cinder primarily composed of glass with some crystalline material. Images were captured on both dry material and ice-grain mixtures. Icy samples are prepared using a thermal sintering protocol, which has been shown to produce the most realistic approximation of microstructure (*20*), and imaged after 5 thermal cycles to reach textural equilibrium and images are captured at −40°C.

Grain packs have a bulk porosity of approximately 37%, which is comparable to the porosity of the lunar regolith below 5 m depth as measured by the Chang’E-3 Lunar lander (*21*). Most void space is large pores between grains, but the grains themselves are also porous. These pores tend to be small, on the order of several microns, and unconnected to other porosity. They likely do not represent flow channels, but may be sights of trapped volatiles. Bulk pore space is partially filled with ice, which exists both along grain boundaries and within bulk pores. Distribution of ice within the sample is heterogeneous, with some dry pockets and local ice fractions up to 90%. Details of image segmentation and analysis can be found in the Supplementary Material.

The segmented microtomography data indicate that this microstructure is both isotropic and relatively homogeneous across grains, ice, and pore space. As shown in fig. S5, A to C, aspect ratio distributions for all three phases are broad but largely overlapping, with most objects clustering around moderate values (∼0.45–0.55) and without evidence for a strongly elongated or directionally biased population, suggesting no dominant shape anisotropy. Likewise, orientation histograms (fig. S5, D to F) are approximately flat across −180° to 180°, with no persistent preferred alignment beyond minor statistical spikes, consistent with random phase orientations and therefore isotropic fabric. Together, the absence of strong directional peaks in theta and the similar morphological distributions among phases support a microstructure lacking large-scale fabric organization or spatial segregation, consistent with a homogeneous, isotropic starting material. While large uncertainty remains regarding the texture of ice in the lunar regolith, these images give us a first order estimate of how ice can exist within regolith packs and help inform our rock physics models.

### Thermal model

In developing a model of seismic velocity through icy materials, we first develop a realistic model of potential ice distributions at and near the lunar surface. These models are adapted from a family of ice stability models originating from (*22*), later applied to Mercury (*23*), the Moon (*24*, *25*) and potential lunar landing sites. These models use ray tracing to monitor radiation exchange between triangular facets based on real lunar terrain. Each facet is underlain by a 100-layer finite element thermal model representing the upper 1.5 meters of the lunar surface. Below this depth, diurnal variations in temperature are minimal, resulting in constant temperatures, here treated as a heat flux at the bottom boundary. For this surface model, lateral thermal conduction is so small that it is neglected.

**Fig. 2.**
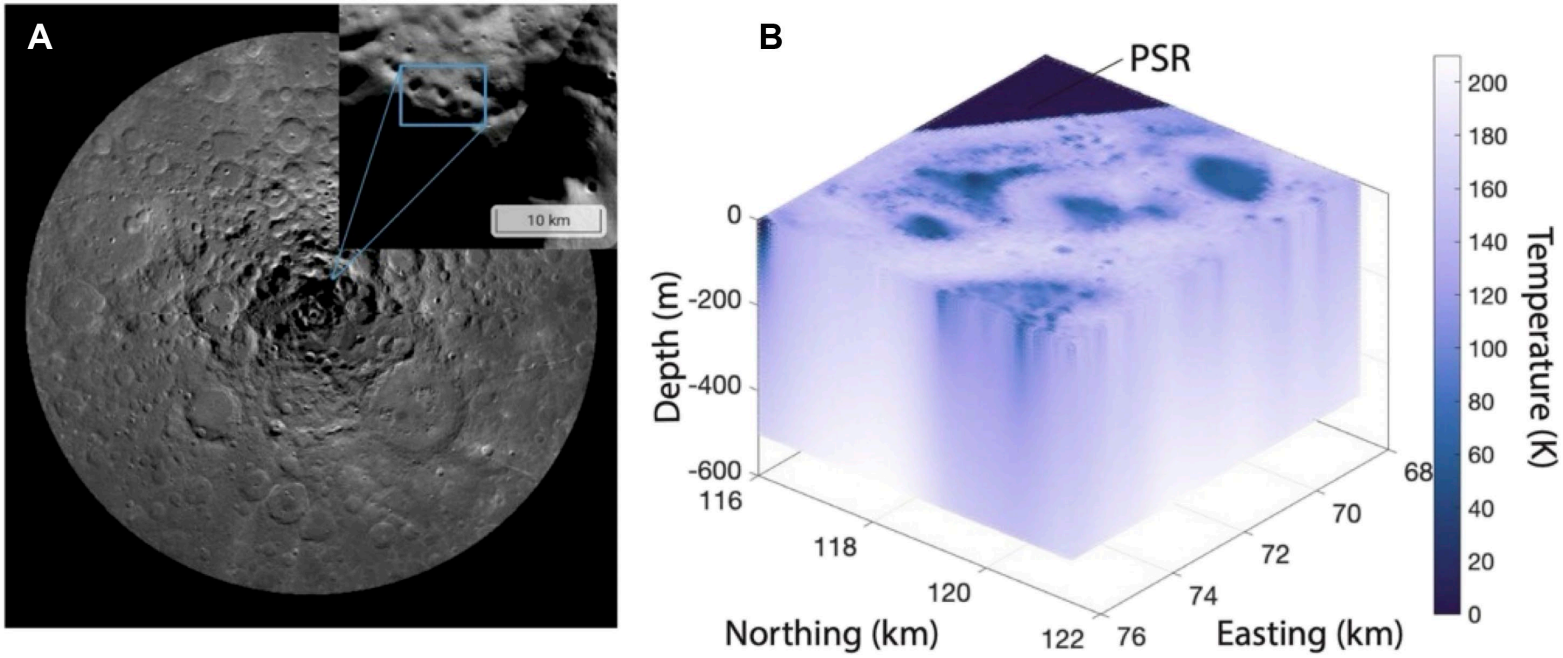
Thermal model of Mons Mouton. Map of modeled region. (**A**) Photograph of lunar south pole. Background imagery from LROC NAC/WAC data visualized using the LROC QuickMap tool. LROC data from Robinson *et al.* (2010) (*50*). (**B**) Thermal model of the temperature structure of the Mons Mouton location near the lunar south pole. Projection is polar stereographic centered on coordinates −85.4096 deg. S, 31.1630 deg. E.

Thermal properties follow (*26*) and heat flux is assumed as 15 mW/m^2^. Here we present a nominal model for the Mons Mouton region of the Moon (Fig. 2), the landing site target for the Volatiles Investigating Polar Exploration Rover (VIPER) lunar rover mission (*27*). This area is known for its abundance of permanently shadowed regions, likely candidates for persistent ice (*28*). The extremely low conductivity of the lunar regolith creates extremely stable temperatures at 1.5 m depth in this region, with stable temperatures that do not vary over the diurnal surface temperature swings. Large areas where ice should remain stable from sublimation over geologic times (>1–2 Gyr) are found in multiple ∼100 m diameter craters, motivating this region as a site for volatile exploration. Loss rates will decrease to ∼1 m/Gyr at temperatures below roughly 100 K at the surface or as high as 145 K when buried below several meters of regolith (*29*).

### Velocity model

**Fig. 3.**
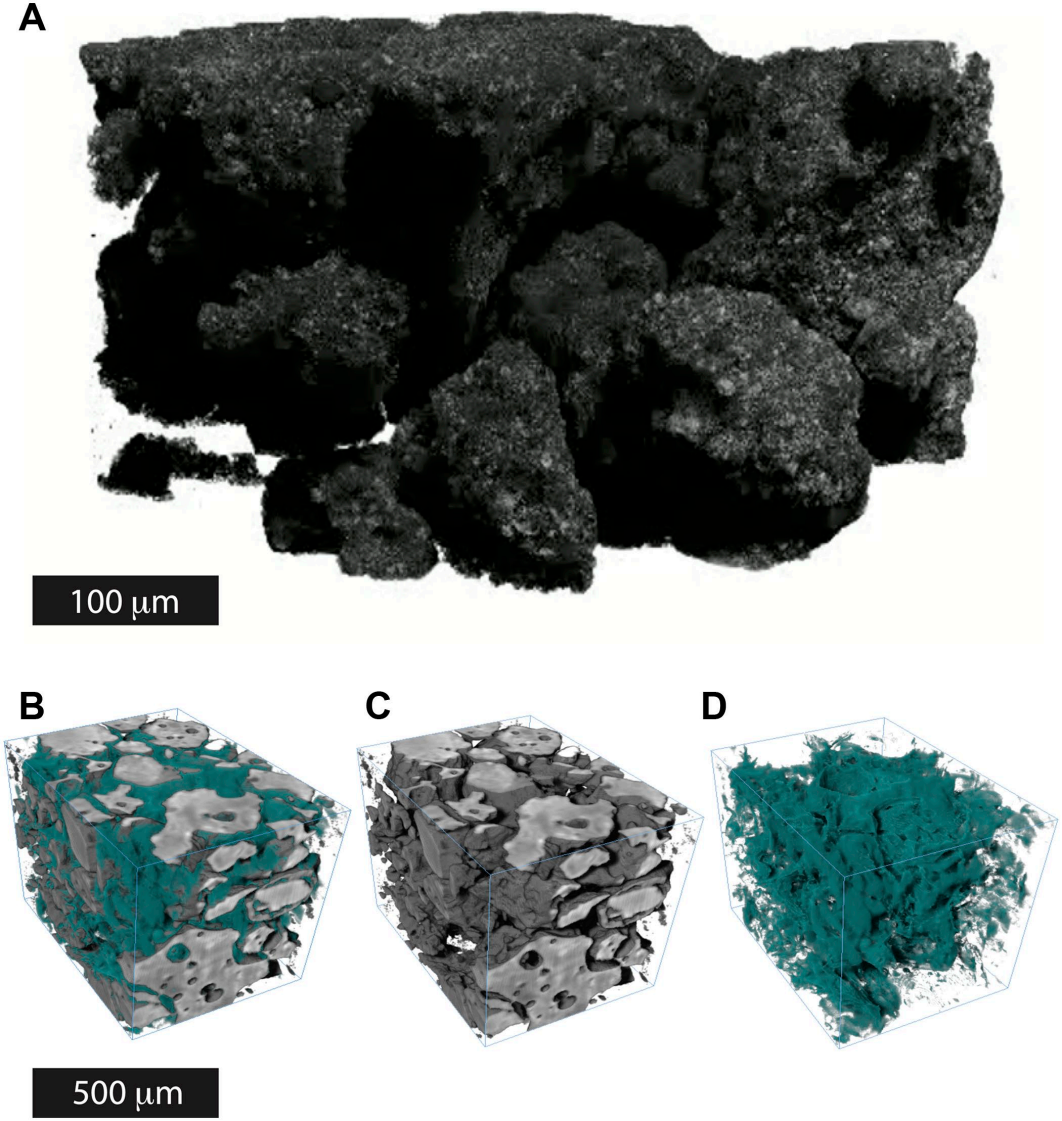
Volume renderings of icy regolith microstructures. Data from x-ray tomography of regolith simulant JSC-1A. (**A**) High resolution scan of dry starting materials highlights bright crystallites in dark glassy matrix. (**B**) Rendering of icy microstructures from sample prepared with 4 wt% water. (**C**) Grains with the ice removed and (**D**) ice with the grains removed. Ice exists as both grain boundary cement and isolated within pore space.

**Fig. 4.**
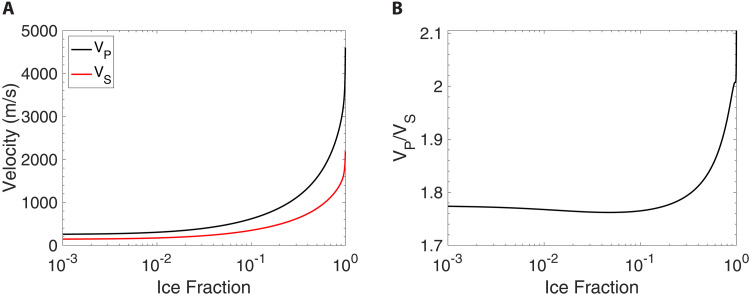
The effect of ice fraction on regolith velocity. Modeled seismic velocity as a function of ice fraction for the H-S grain boundary geometry. (**A**) Compressional (V_P_) and shear (V_S_ ) velocity and (**B**) the V_P_/V_S_ ratio for increasing ice fractions.

Once we have calculated the density and the bulk and shear moduli as a function of ice fraction, we can construct a seismic velocity model to link measured velocities to varying ice content and assumed structures. Selection of the correct rock physics model is essential to capture the true behavior of a material. Microstructural analysis of the tomography data indicated the microstructure of the icy regolith grain pack is complex, exhibiting multiphase grain contacts and diverse contact geometry; this is best represented by the Hashin-Shtrikman average as the assumptions of this model most closely fit our sample microstructure (Fig. 3) and it is often used successfully to model complex sediments (*30*, *31*). The results of this velocity model are presented in Fig. 4.

**Fig. 5.**
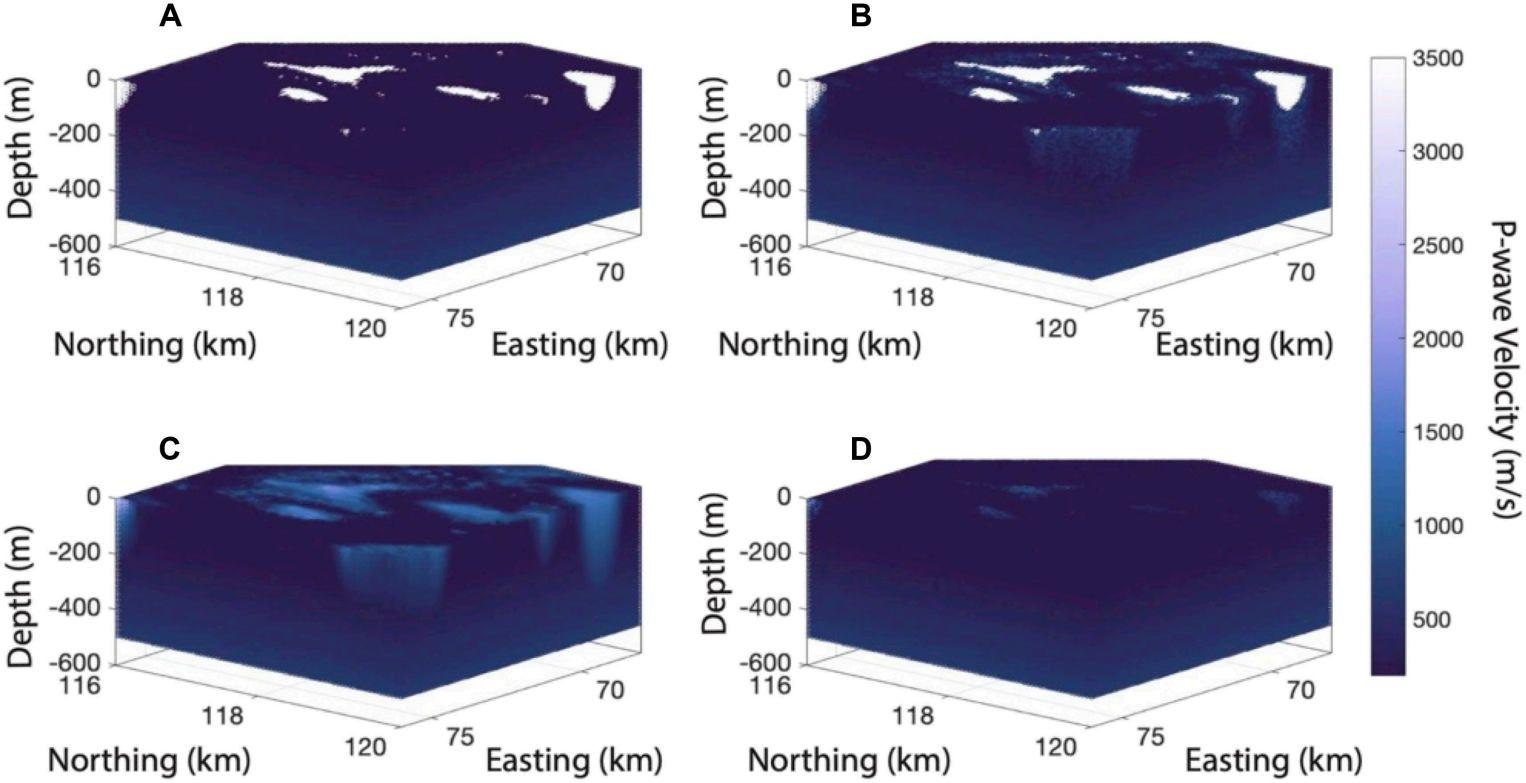
Velocity models of Mons Mouton. Four realizations of seismic velocity models with variable ice content. (**A**) 6.2 wt% ice frozen into regions with subsurface temperatures <140 K. (**B**) 7.6 wt% ice, frozen into pore space under 80 K with disseminated ice in pore space between 80 and 140 K. (**C**) 6.0 wt% ice, all pore space below 140 K with disseminated ice, and (**D**) 1.3 wt% ice, all pore space below 80 K with disseminated ice.

We use these velocity-ice fraction relationships to populate several theoretical 3D velocity models of the lunar south pole (Fig. 5). The source, longevity, transport and distribution of volatiles in the regolith remain active areas of research (*32*, *33*), so we have made four realizations of the model using different assumptions to test a range of possible distributions. We start with a background density and velocity model using the depth profiles of (*34*–*36*), respectively. A 1D profile of the starting velocity model is presented in fig. S12. We then added ice content to the model using our thermal model as a guide. Schorghofer (*29*) has modeled ice longevity up to 140 K in dust covered regolith, so we used this cutoff as a guide for our different realizations. In the first realization (Fig. 5A), we filled all pore space below 140 K with ice, creating craters with frozen floors. In the second realization (Fig. 5B), we filled pore space under 80 K with ice and populated ice content stochastically between 0 and 1 in pore space between 80 and 140 K with content inversely proportional to temperature, creating craters with frozen floors and ice haloes. In the third realization (Fig. 5C), we populated all pore space below 140 K with stochastically variable ice content inversely proportional to temperature, creating ice haloes that center on the craters in the region. In the fourth realization (Fig. 5D), we populated all pore space below 80 K with stochastically variable ice content inversely proportional to temperature, creating randomly distributed ice only within the crater floors (a muted version of 5C). Ice content was then converted to seismic velocity using our model from Fig. 4. It should be noted that although there are locally high areas of high ice content, the total weight percent of water in the models varies from 2–8%, in agreement with LCROSS measurements (*2*).

### Synthetic seismic experiment

**Fig. 6.**
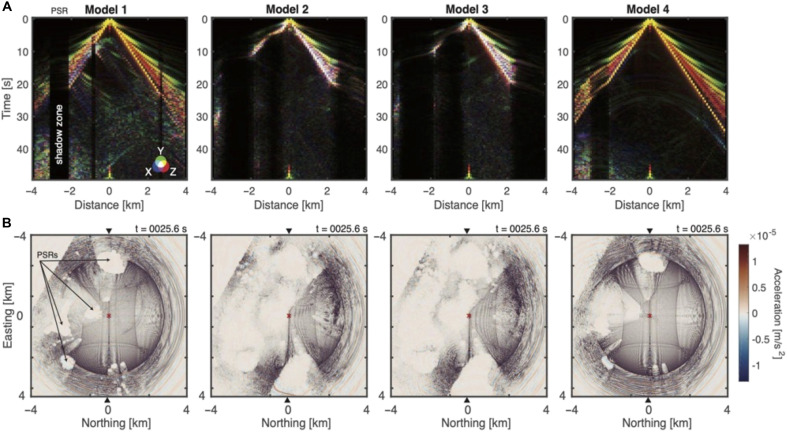
Synthetic seismic models. Results from the seismic simulations for models of ice; models are described in Fig. 5. (**A**) Polarization of the wavefield for a line of west-east stations placed every 100 meters from −4 to 4 km across the array, as indicated by the triangles in the wavefield plots in part (B). The colormap in the lower left shows the relative direction of motion, components are normalized to the peak displacement in the wavefield. (**B**) Wavefield snapshots taken at 25.6 seconds into each simulation. Locations of large PSR regions are highlighted with arrows, the source is indicated with a red cross. Triangles indicate the location of the seismometer array placed across the simulation and shown in part (A). The waveforms for the vertical component of motion are plotted in figs. S7–S10.

The four seismic velocity models of ice structure in the Mons Mouton region are then used as background models for a set of 3D seismic wave propagation finite difference simulations to study the effects of ice infused regolith on the seismic wavefield (Fig. 6). The Apollo missions conducted both passive (*37*) and controlled source seismic experiments (*38*, *39*) on the Moon, which found evidence for a highly scattered and diffuse wave propagation (*40*, *41*). Our goal here is not to reproduce the exact waveforms of Apollo observations, but rather to study how isolated regions of heterogeneity imposed by ice would affect waves, and map to the more scattered wavefields of the Moon. Thus, we do not incorporate random media into our background model. We examine the resulting wavefields in two ways; one through a seismic array with three-component seismometer stations placed every 100 meters across the box (Fig. 6A), and through snapshots of the wave interactions with the ice-infused regolith (Fig. 6B). The 3-component wavefield is visualized by assigning intensity to amplitude, and red (vertical), green (radial), and blue (transverse) motions. Regions that appear white is where scattering has depolarized the waves.

Amplitude effects are evident in the simulations and dependent upon the geometry of the ice. For the classes of models 1 and 2 (Fig 5, A and B) with regions of contiguous ice, the higher seismic velocities of the ice-infused regolith produce a near total reflective boundary, with reflection coefficients of >0.9 for P and S waves. This results in over 90% of the seismic amplitude being scattered from the ice-infused region as a back-scattered reflection (seen as a backward propagating wave arriving 5–10 seconds later in the station). The high reflection coefficient results in a seismic shadow zone for a sensor placed in any of the low-temperature permanently shadowed regions. Depending on the model, amplitudes within the ice-infused region are 10–20% of the surrounding ice-free zones. Energy detected on the other side of the ice-infused regions is from diffraction around the edges and bottom of the icy region, these waves reappear on the other side of craters with diminished amplitudes. These shadow zones extend into any region with diffuse ice and a 3D survey of shadow zones are diagnostic of ice presence. In models where a random halo of ice is introduced into the subsurface (Fig. 5, B and C, models 2 and 3 respectively), the wavefield becomes progressively more scattered, which is evident in the polarization plot of Fig. 6, where the amplitudes whiten where surface wave energy is scattered. Ice models 2 and 3 produce the largest attenuation of the seismic wavefield, with a coda of depolarized energy and extensive shadow zones across 75% of the seismic profile. Only the zone to the north with less ice has a wavefront. Notably, the model with the least amount of distributed ice (model 4) still exhibits a muted shadow zone within the western region, elevated velocities in the region of ice, and minimal scattering to the east where few ice anomalies exist.

We also examine the sensitivity of the wavefield to ice presence. Measuring the first arriving P-waves and the moveout for S, and surface waves provides the average velocities of the medium. In all cases, any wave traveling in the ice-infused regions have velocities >1000 m/s, a factor of 2–3 higher than the surrounding regolith models. We note that on the Moon any wavefield would be highly scattered by impact-induced heterogeneity in the regolith, but in general the seismic arrival times are conserved (*38*, *42*). The waves traveling in the ice-infused regions move faster than the waves in surrounding dry regolith; the higher the ice abundance, the faster the wave. Measurement of the regolith velocity between two stations placed in a PSR vs those without would be indicative of whether ice was present, and the change in velocity would be indicative of ice abundance (e.g., 3500–4000 m/s in model a; 1000 m/s in model d). Regions without ice would be expected to have smaller variations in seismic velocity between stations.

## DISCUSSION

Our integrated investigation demonstrates that the seismic signature of lunar regolith is strongly modulated by the presence and distribution of ice. By combining high-resolution synchrotron x-ray microtomography, effective medium modeling, and advanced seismic simulations, we have shown that even modest ice fractions can induce meaningful changes in scattering behavior and wave velocities. The application of effective medium theories provides robust constraints on the elastic properties of ice-infused regolith, in agreement with prior work on heterogeneous materials (*15*, *30*). Our results indicate that variations in seismic behavior can serve as reliable indicators of both the presence and the abundance of subsurface ice. This suggests that seismic exploration, long used in terrestrial resource prospecting, holds promise for mapping lunar volatile deposits.

The implications of these findings extend beyond resource identification; they contribute to our broader understanding of lunar geophysics. The altered seismic properties resulting from ice presence not only facilitate the detection of water but also influence the overall mechanical behavior of the regolith. Such insights are critical for designing lunar infrastructure and planning future exploration missions, as well as for comparative planetology studies that consider ice stability on other airless bodies (*23*).

The effective medium models employed assume a level of microstructural homogeneity that may oversimplify the inherent complexity of the lunar regolith, where anisotropy and heterogeneous phase connectivity likely play important roles, but ongoing efforts to better characterize these materials are likely to yield substantial improvements to this limitation. Replication of the extreme vacuum, thermal cycling, and space weathering present on the lunar surface during experiments will further advance characterization efforts. Our seismic simulations also omit factors such as realistic topography and multi-scale scattering effects, which influence wave propagation (*40*, *43*); however, these effects are not necessary to illustrate how ice-infused regolith would deviate from the typical seismic wave propagation in the Moon. Any assumption of random heterogeneity would produce a seismic coda in the models that would be modulated by the ice-model heterogeneity. The primary wavefield effects of shadow zones and increased velocity would remain in simulated data that incorporate realistic scattering. Adding this complexity to higher resolution modeling efforts utilizing higher frequency sources such as those proposed for future exploration missions (*44*) would facilitate more robust results.

Moving forward, additional work is needed to extend microstructural analyses to a wider array of regolith analogs and to develop more sophisticated models that account for anisotropy and heterogeneity. Enhanced seismic simulations that incorporate realistic topographic features, complex scattering mechanisms and detailed near-surface structure information will be vital. Collaboration with upcoming lunar missions—such as those deploying advanced seismometers (*45*) and integrated multi-sensor platforms (*46*) will be crucial for validating these models under actual lunar conditions.

We propose that a set of seismometers placed inside and outside a PSR, coupled with a controlled seismic source initiated either inside or outside the PSR would allow distinction between various ice-regolith scenarios and ice distribution. Nearby passive sources would also provide a comparison of wave propagation effects. Such sources would enable measurement of the regolith seismic velocity within and without the PSR region, with elevated velocities indicative and sensitive to ice abundance. Although other mechanisms could increase seismic velocity variations (e.g., the presence of frozen melt sheets or lava flows), the spatial relationship of seismic velocity to where lowered temperature exists would be strong evidence for buried ice resources and provide a tool for prospecting the deposit. Detection by the sensors of a shadow zone associated with a PSR without loss of source polarization would be indicative of tightly bound ice with sharp transitions existing between regions of ice. Depolarization of the source wavefield and loss of amplitude by the station within the PSR would be evidence for widely distributed and diffuse ice vs a sparser geometry of ice in the surrounding regolith. Scattering effects for lunar seismic waves are more circumstantial, as scattering can be produced by non-ice related heterogeneity within the regolith and megaregolith. However, like for seismic velocity, if scattering effects are localized on a particular PSR, then the pattern could be used to isolate the presence of ice. Advanced 3-D surveys and larger numbers of seismic instruments could follow and characterize the resource further. New results from the recent Chandrayaan-3 deployed seismometer (*45*), upcoming missions to the lunar farside [the Farside Seismic Station; (*47*)] and the Lunar Environmental Monitoring Station for Artemis III [LEMS, (*48*)] near the south pole will provide opportunities to seismically sample the south polar subsurface.

Our findings provide a promising framework for the seismic detection of lunar ice, offering valuable insights into the mechanical and geophysical behavior of volatile-infused regolith. Continued experimental and modeling efforts will be essential to fully translate these findings into reliable exploration strategies for future lunar missions.

## MATERIALS AND METHODS

### Ultrasonic measurements

For the saturated sample, an ultrasonic pulse-transmission system consisting of an upright cylindrical acrylic column (internal diameter, 107.5 mm and height, 150 mm) outfitted with ultrasonic and temperature sensors. The two transducer ports, one for the transmitter and the other for the receiver, were positioned at opposite sides about the symmetry axis of the column, so that the acquired signals propagated along the diameter of the cylindrical sample; Both P- and S-wave transducers were used. A square-wave pulse generator (Panametrics 5077PR) was used to provide high-voltage (100–400 V), high-frequency (central frequency 1 MHz) excitation signals for the ultrasonic transducers. To achieve good acoustic coupling between the transducers and the saturated samples, immersion-type transducers (central frequency, 1 MHz; Panametrics V303-SU) were used. The transmitted ultrasonic signals were acquired using a digital oscilloscope (Tektronix MDO3014) and saved on a control PC for analysis. Schematic and images of the ultrasonic cell are presented in fig. S1. For each sample, the regolith material was first weighed then poured into the testing cylinder. Samples were agitated by tapping to settle grains, tamped, then grain pack height was measured. For the water saturated sample, distilled water was then poured to the top of the grain pack and agitated again to remove air bubbles. Two parallel experiments were run. The first was in a fully water-saturated sample and the second was a miniaturized version of the ultrasonic cell with a nominally dry sample. The nominally dry sample was not oven-dried and there was no attempt made to drive off adsorbed water, so that the surfaces of the grains were hydrated from ambient atmospheric humidity. The water-saturated sample was taken to −10°C and the dry sample was taken to −40°C, the limits of the individual freezers used. The larger freezer cannot achieve as cold temperatures, but has greater temperature stability. Ultrasonic measurements were then taken as the samples were slowly warmed. Each temperature step was given at least 24 hours to stabilize.

### Synchrotron x-ray microtomography

Synchrotron x-ray microtomography was performed at beamline 8.3.2 at the Advanced Light Source (ALS). The sample was scanned using monochromatic light at 32 keV and an optical chain consisting of a 500 mm Ce-doped LuAG scintillator (Crytur), 2× and 10× Mitutoyo objective lens with long working distance (0.055 numerical aperture), and a pco.edge 2560 by 2160 pixel sCMOS detector, resulting in pixel sizes of 3.5 and 0.65 microns with lateral FOVs of 5 and 0.9 mm.

### Thermal modeling

Starting with the thermal model of Siegler *et al.* (*24*), we extend the model deeper than 1.5 m by considering lateral heat conduction. As density increases, the near surface thermal conductivities of ∼1e-3 W/m/K increase to values of ∼0.1 W/m/K or greater at depth. As a first order model of lateral heat conduction at depth, we apply our 1.5 m temperature to a 3D finite element mesh. This is done using Comsol Multiphysics, which provides an irregular mesh with ∼1 m element resolution. Thermal conductivity of this deeper layer is assumed to be a constant 0.1 W/m/K, or densities of roughly 1 kg/m3. We extend this model to 500 m depth. While densities and therefore thermal conductivities likely increase over these values at depth, this provides a first order estimate of how deep an area of permanent shadow.

### Seismic modeling

The seismic simulations are performed with Wave Propagation Program (*49*), which uses a 2nd order finite difference scheme on a cartesian and curvilinear mesh designed for efficient wave simulations in parallel high end computing environments. The simulation area is 8000 by 8000 m and extends 1000 m into the subsurface, with a 4 meter grid spacing. For the lowest wavespeeds in the simulation, (116 m/s) this provides ∼30 points per wavelength, and allows us to compute a wavefield accurate to 3.5 Hz, overlapping the sensitivity of the past Apollo experiments (*8*). We place a strike slip moment tensor source at the center of the simulation, and generate a moonquake with a Mw = −4.5 magnitude, and focal mechanism with a strike = 0°, dip = 35°, and rake = 90° at 10 meters depth, and corner frequency of 5 Hz. The simulation is run out to 50 seconds at which time the waves begin to interact with the edges of the box creating non-physical reflections at the boundaries. Sensors are placed through the simulation, with snapshots of the wavefield collected every 1 second in the simulation. Note that we do not implement topography or random media scattering beyond the ice models into the simulation, although these properties likely affect wave propagation on the Moon (*41*), as we wish to isolate the effects of the ice on our wave propagation.
