# Supplementary material for: The seismic signature of lunar ice
Source: Sci Adv. 2026 Jul 31;12(31):eadz7220. doi: 10.1126/sciadv.adz7220 (PMC13426401; doi:10.1126/sciadv.adz7220)
Supplement: Supplementary file 1 — Supplementary Text Figs. S1 to S12 [file sciadv.adz7220_sm.pdf]

Science Advances
AAAS

Supplementary Materials for

The seismic signature of lunar ice

Harrison P. Lisabeth *et al.*

Corresponding author: Harrison P. Lisabeth, hlisabeth@lbl.gov

Sci. Adv. **12**, eadz7220 (2026)
DOI: 10.1126/sciadv.adz7220

This PDF file includes:

Supplementary Text
Figs. S1 to S12

Supplementary Text

Ultrasonic measurements

We measure elastic velocities using ultrasonic sensors in transmission geometry (Supplementary Figure S1). Waveforms were successfully recovered at all temperatures, but amplitudes tended to be very low above freezing. Collected waveforms are shown in Supplementary Figure S2.

The largest variations are between the liquidus and solidus temperature for ice (Figure 3). The saturating fluid here was distilled water, but we cannot rule out traces of salts on the starting material. The solidus temperature appears to be very near 0°C. A few key observations are that the saturated sample is faster than the dry and wet samples at all conditions. The P/S ratios of the saturated samples show clear temperature trends in both velocity and amplitude and the wet sample has greater P-wave velocity variations than the saturated sample.

Rock physics models

Below are the mathematical expressions for the Reuss, Voigt, Voigt-Reuss-Hill (VRH), and Hashin-Shtrikman models, used to estimate the effective elastic properties of composite materials. These models provide estimates for the bulk modulus, K, and shear modulus, G, of a composite based on the properties of its constituent phases.

1. Reuss Model

The Reuss model assumes that all phases are under the same stress (isostress condition). It gives the lower bound of the effective moduli for a composite.

Bulk Modulus (Reuss):

$$\frac{1}{K_R} = \sum_i \frac{f_i}{K_i}$$

where f_i is the volume fraction of phase I and K_i is the bulk modulus of phase i.

Shear Modulus (Reuss):

$$\frac{1}{G_R} = \sum_i \frac{f_i}{G_i}$$

where G_i is the shear modulus of phase i.

2. Voigt Model

The Voigt model assumes that all phases experience the same strain (isostrain condition). It gives the upper bound of the effective moduli for a composite.

Bulk Modulus (Voigt):

$$K_V = \sum_i f_i K_i$$

Shear Modulus (Voigt):

$$G_V = \sum_i f_i G_i$$

3. Voigt-Reuss-Hill (VRH) Average

The VRH model is the arithmetic mean of the Voigt and Reuss bounds, often used as a practical approximation for the effective elastic moduli of composites.

Bulk Modulus (VRH):

$$K_{VRH} = \frac{K_V + K_R}{2}$$

Shear Modulus (VRH):

$$G_{VRH} = \frac{G_V + G_R}{2}$$

4. Hashin-Schtrikman (HS) Bounds

The Hashin-Schtrikman bounds give more tightly constrained upper and lower limits for the effective moduli of a composite by taking into account the interaction between different phases and assuming random distributions of phases with isotropic properties. HS assume a random, statistically isotropic, heterogeneous medium composed of isotropic phases such that the medium can be considered homogeneous on the scale of the imposed strain, and they derive the tightest possible bounds on the effective elastic moduli consistent with these assumptions. For a composite of two phases (f1 and f2), the HS bounds for bulk modulus, K, and shear modulus, G, are given by the following expressions:

Upper Bound for Bulk Modulus (HS Upper):

$$K_{\mathrm{HS}}^{\mathrm{upper}} = K_2 + \frac{f_1}{\frac{1}{K_1 - K_2} + \frac{(1-f_1)}{K_2 + \frac{4}{3}G_2}}$$

Lower Bound for Bulk Modulus (HS Lower):

$$K_{\mathrm{HS}}^{\mathrm{lower}} = K_1 + \frac{f_2}{\frac{1}{K_2 - K_1} + \frac{(1-f_2)}{K_1 + \frac{4}{3}G_1}}$$

Upper Bound for Shear Modulus (HS Upper):

$$G_{\mathrm{HS}}^{\mathrm{upper}} = G_2 + \frac{f_1}{\frac{1}{G_1 - G_2} + \frac{2(1-f_1)(K_2 + 2G_2)}{5G_2(K_2 + \frac{4}{3}G_2)}}$$

Lower Bound for Shear Modulus (HS Lower):

$$G_{\mathrm{HS}}^{\mathrm{lower}} = G_1 + \frac{f_2}{\frac{1}{G_2 - G_1} + \frac{2(1-f_2)(K_1+2G_1)}{5G_1(K_1+\frac{4}{3}G_1)}}$$

An arithmetic average of the Hashin-Schtrikman (HS) bounds can be taken in a similar way as the Voigt-Reuss-Hill (VRH) average, which is the arithmetic mean of the Voigt and Reuss bounds. For the HS bounds, you would average the upper and lower HS bounds for both the bulk modulus,K, and the shear modulus, G.

Arithmetic Average of the HS Bounds for Bulk Modulus:

$$K_{\mathrm{HS,avg}} = \frac{K_{\mathrm{HS}}^{\mathrm{upper}} + K_{\mathrm{HS}}^{\mathrm{lower}}}{2}$$

Arithmetic Average of the HS Bounds for Shear Modulus:

$$G_{\mathrm{HS,avg}} = \frac{G_{\mathrm{HS}}^{\mathrm{upper}} + G_{\mathrm{HS}}^{\mathrm{lower}}}{2}$$

Microtomography measurements

Synchrotron x-ray microtomography was performed at beamline 8.3.2 at the Advanced Light Source (ALS). The sample was scanned using monochromatic light at 32 keV and an optical chain consisting of a 500 mm Ce-doped LuAG scintillator (Crytur), 2x and 10x Mitutoyo objective lens with long working distance (0.055 numerical aperture), and a pco.edge 2560 pixel x 2160 pixel sCMOS detector, resulting in pixel sizes of 3.5 and 0.65 microns with lateral FOVs of 5 and 0.9 mm.

The high-resolution scan of starting material was captured by mounting the sample in a Kapton tube and placing it in a stage mounted collet. We prepared icy samples by mixing 10 wt% ice grains and with 90 wt% regolith, then placing the mixture in the sample cup for the cryogenic sample chamber (Fig. S4) and the assembly was put in a laboratory freezer. Temperature was then cycled between 0 and -40°C at a rate of 5°/hour 5 times to equilibrate the ice texture. Samples were placed on dry ice to transport from the lab to the beamline. Microtomography images were captured at -40°C.

Image Analysis

Reconstructed microtomography volumes were first processed using machine learning–based segmentation in ORS Dragonfly to classify and separate the primary material phases into grains, ice, and pore space. Following phase segmentation, each phase was further partitioned into discrete multi-region-of-interest (multi-ROI) objects using a watershed-based separation algorithm to isolate individual grains, ice bodies, or pores for quantitative morphological analysis. Shape characteristics were then quantified for each segmented object by calculating aspect ratio from the principal shape tensor, defined here as the ratio of the minimum to

maximum eigenvalues, where a value of 0.5 indicates a spherical or equant morphology and lower values reflect increasing elongation or anisotropy. Orientation was determined from the direction of the principal (maximum) eigenvector, providing a quantitative measure of each object's dominant long-axis alignment within the reconstructed volume. Together, these analyses enabled systematic characterization of phase morphology, elongation, and preferred orientation, providing a quantitative framework for evaluating microstructural anisotropy and fabric development across the sample.

Velocity Models

Velocity models are constructed informed by the thermal model using the rock physics relationship derived from our experimental measurements. Both shear and compressional velocities are modeled. A density model was also created using the ice contents populated from our thermal model using a volume averaging approach.

Supplementary Figures

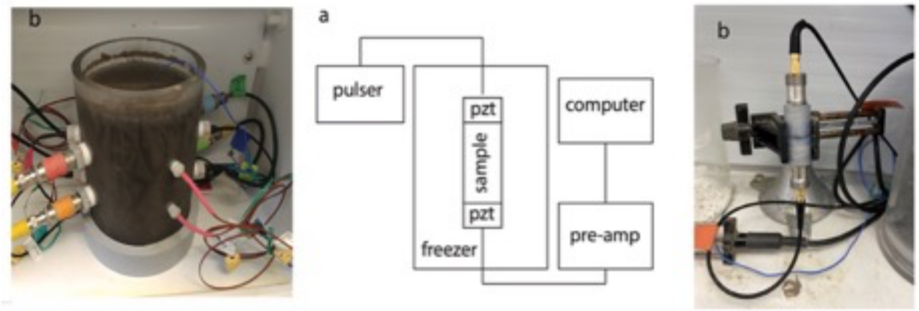

Fig. S1. Experimental apparatus for ultrasonic measurements. a) Schematic of ultrasonic system. b) Photograph of cells in use. Figure modified from (*8*) under the terms of the Creative Commons Attribution 4.0 International License (CC BY 4.0).

.

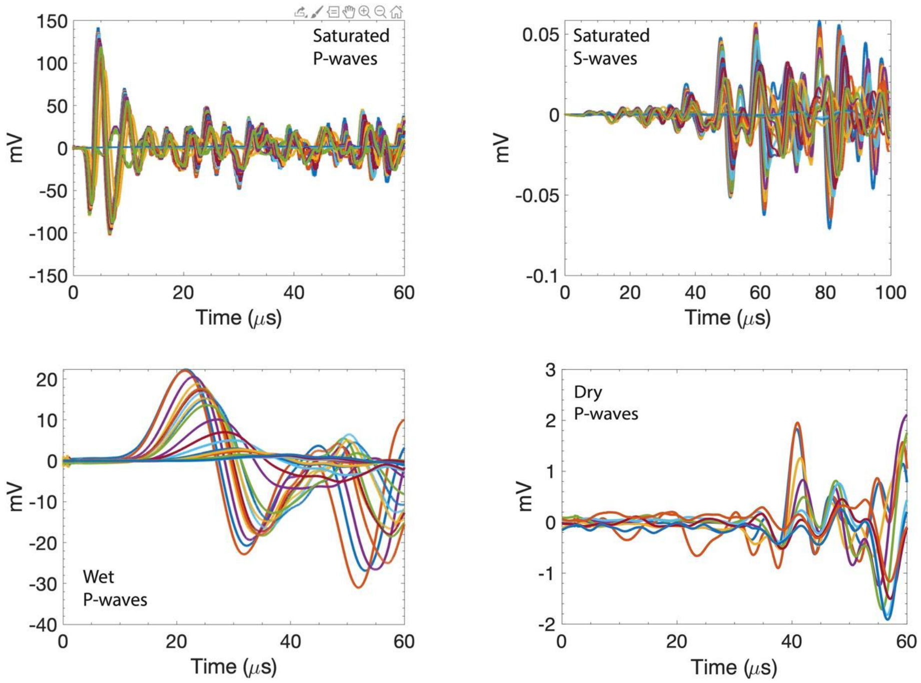

Fig. S2. Waveforms collected from regolith simulant samples. Left to right they are P-waves from nominally dry sample, P-waves from water-saturated sample and S-waves from water-saturated sample. Figure modified from (*8*) under the terms of the Creative Commons Attribution 4.0 International License (CC BY 4.0).

.

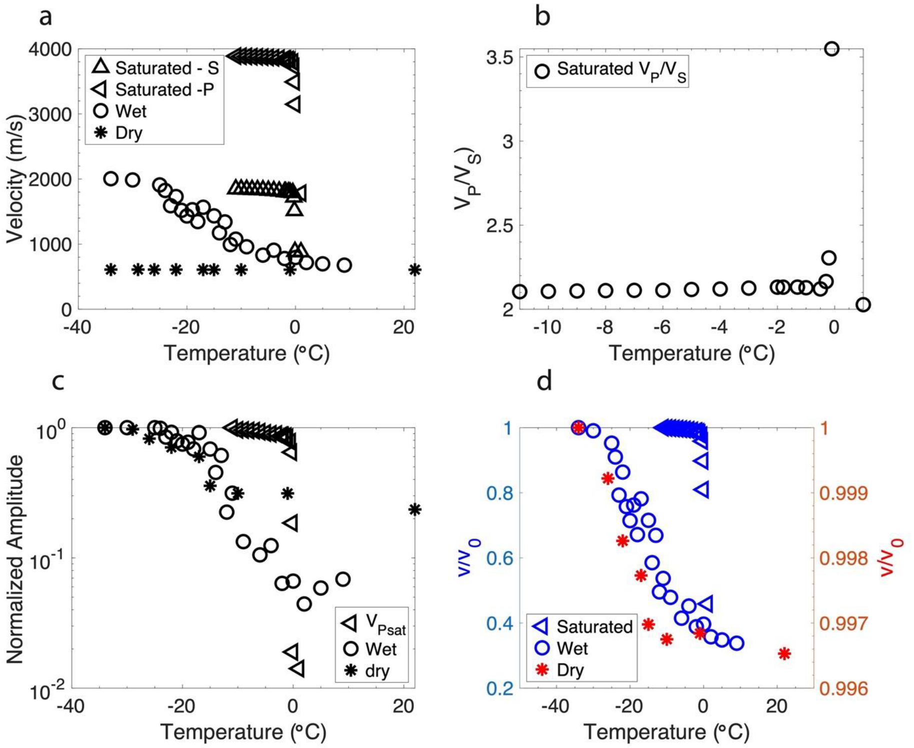

Supplementary Fig. S3. Velocities and amplitudes of waves as they vary with temperature. Amplitudes are normalized to the coldest temperature recorded. Figure modified from (*8*) under the terms of the Creative Commons Attribution 4.0 International License (CC BY 4.0).

.

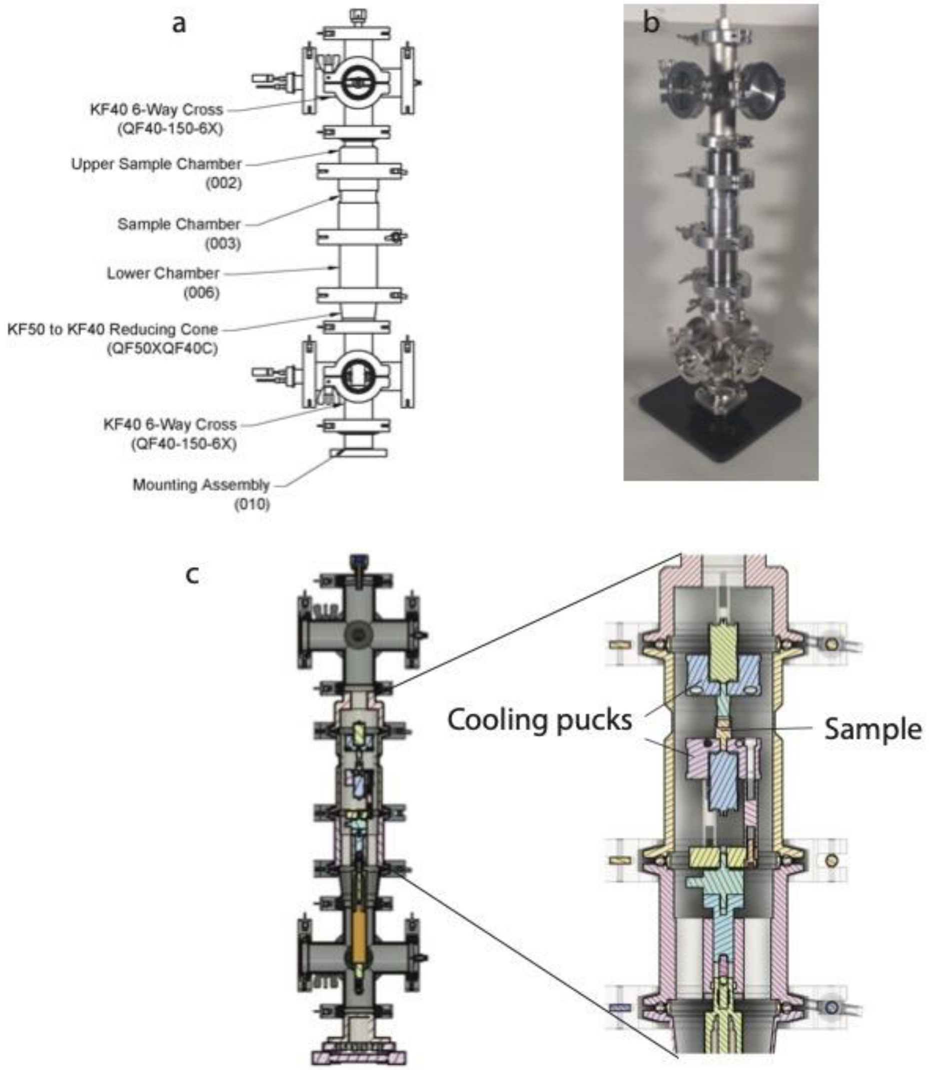

Supplementary Fig. S4. Cryogenic vessel used to image icy regolith samples. a) schematic of vessel, b) photograph, c) exploded cross-section of vessel interior showcasing sample cup and copper cooling pucks.

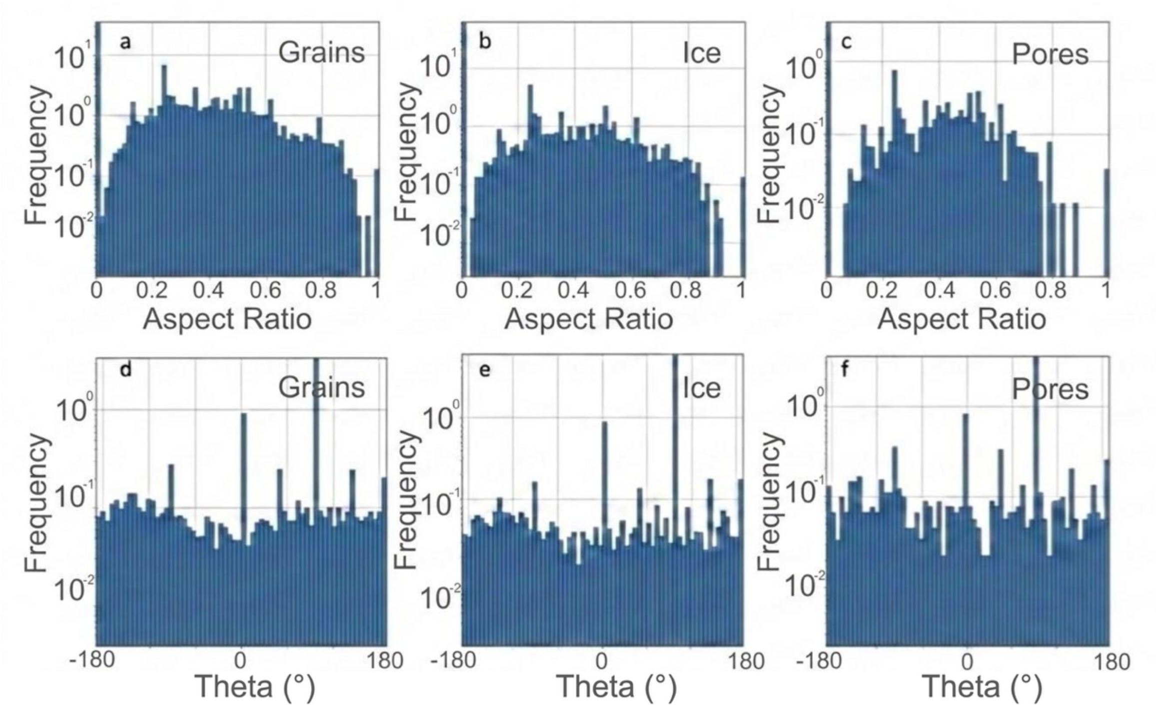

Supplementary Fig. S5. Geometric analysis of icy regolith microstructure. Aspect ratio of a) grains, b) ice and c) pores and shape preferred orientation angle (theta) for d) grains, e) ice and f) pores. No significant grain elongation or orientation is apparent, indicating an isotropic, homogeneous microstructure.

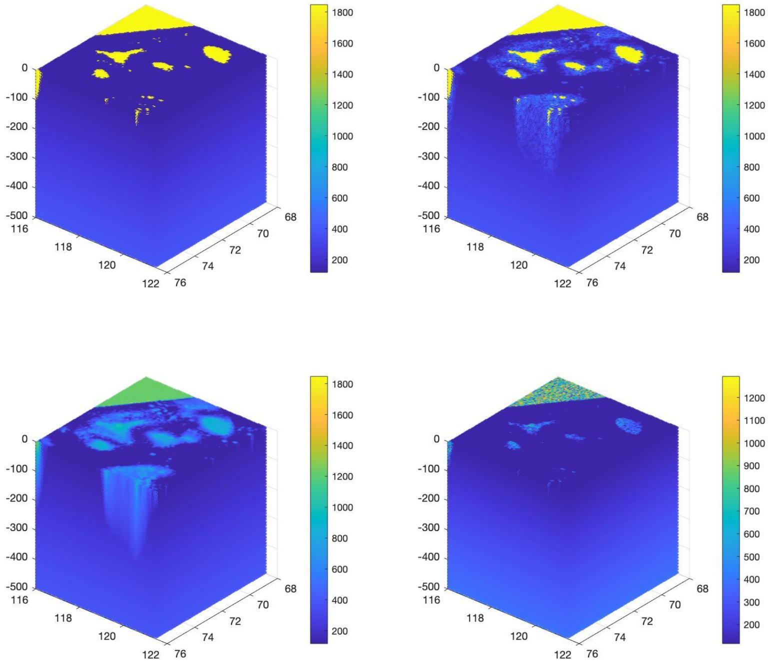

Supplementary Fig S6. Four realizations of our shear velocity (m/s) model with variable ice content.

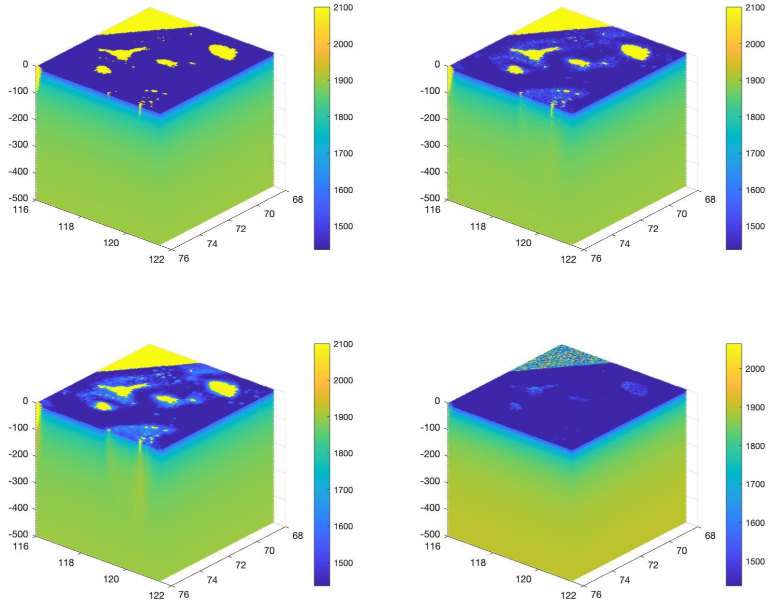

Supplementary Fig S7. Four realizations of our density (kg/m^3) model with variable ice content.

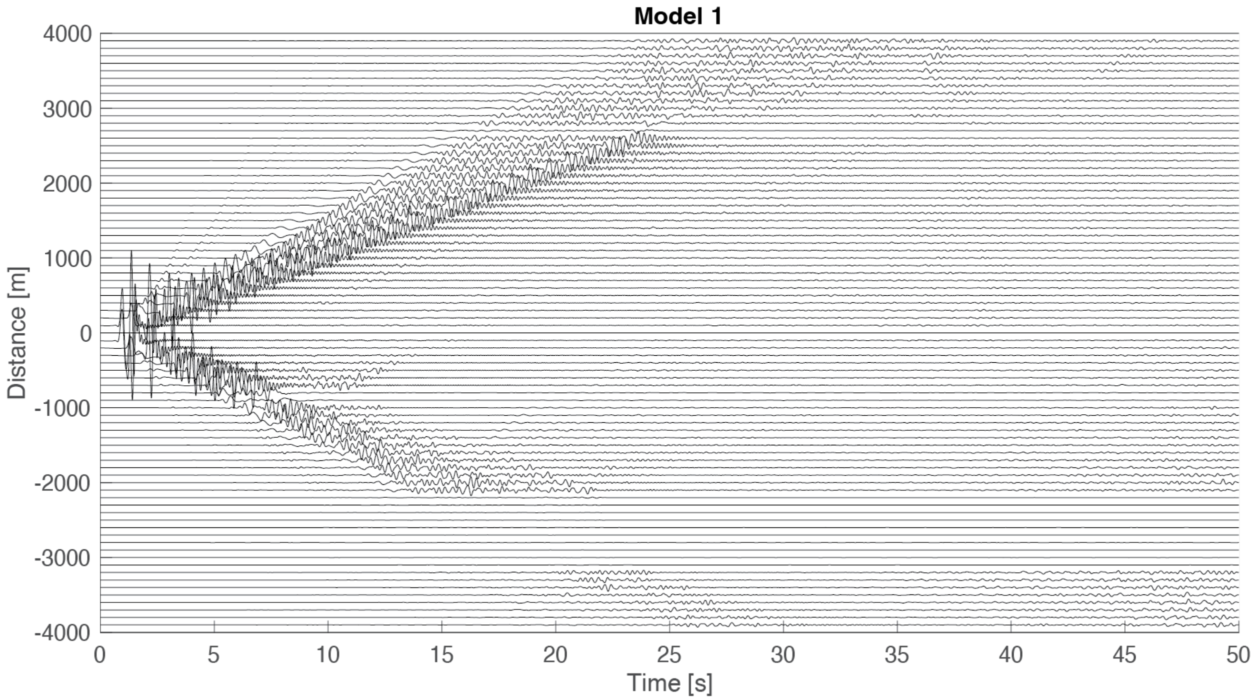

Supplementary Fig S8. Synthetic displacement waveforms for the vertical component of motion for Model 1. Amplitudes are normalized to the peak source and scaled by a factor of 100. The source waveform is not shown as it saturates out of scale.

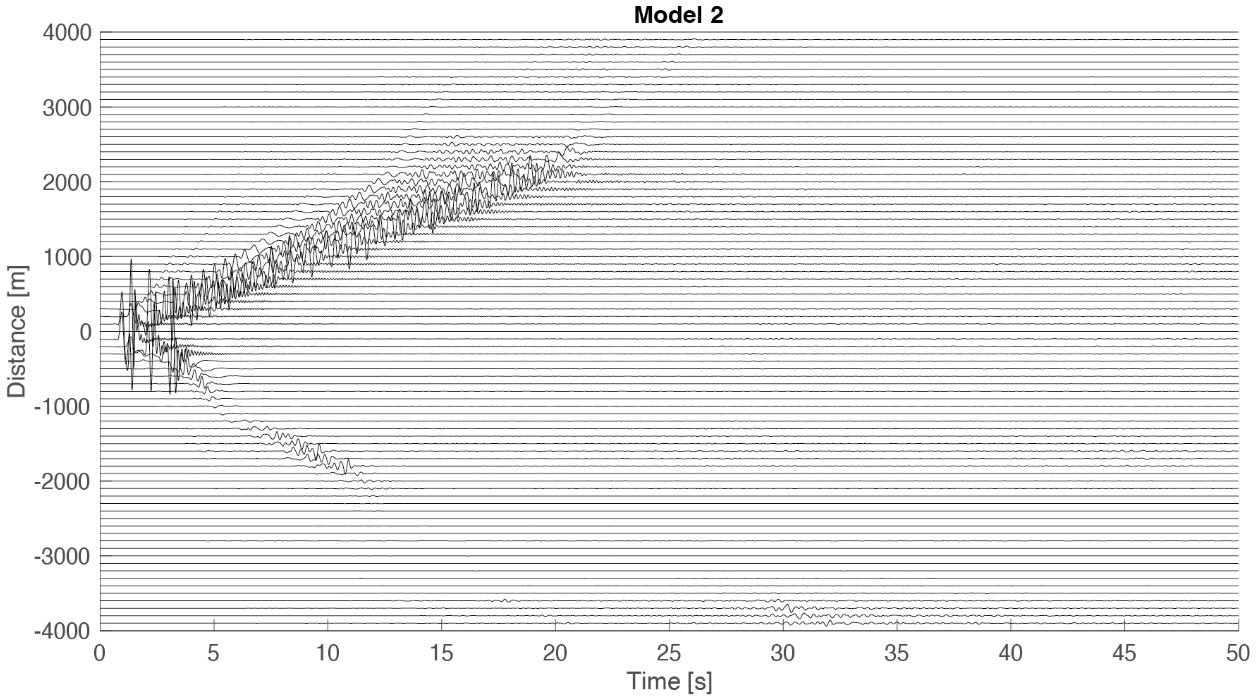

Supplementary Fig S9. Synthetic displacement waveforms for the vertical component of motion for Model 2. Amplitudes are normalized to the peak source and scaled by a factor of 100. The source waveform is not shown as it saturates out of scale.

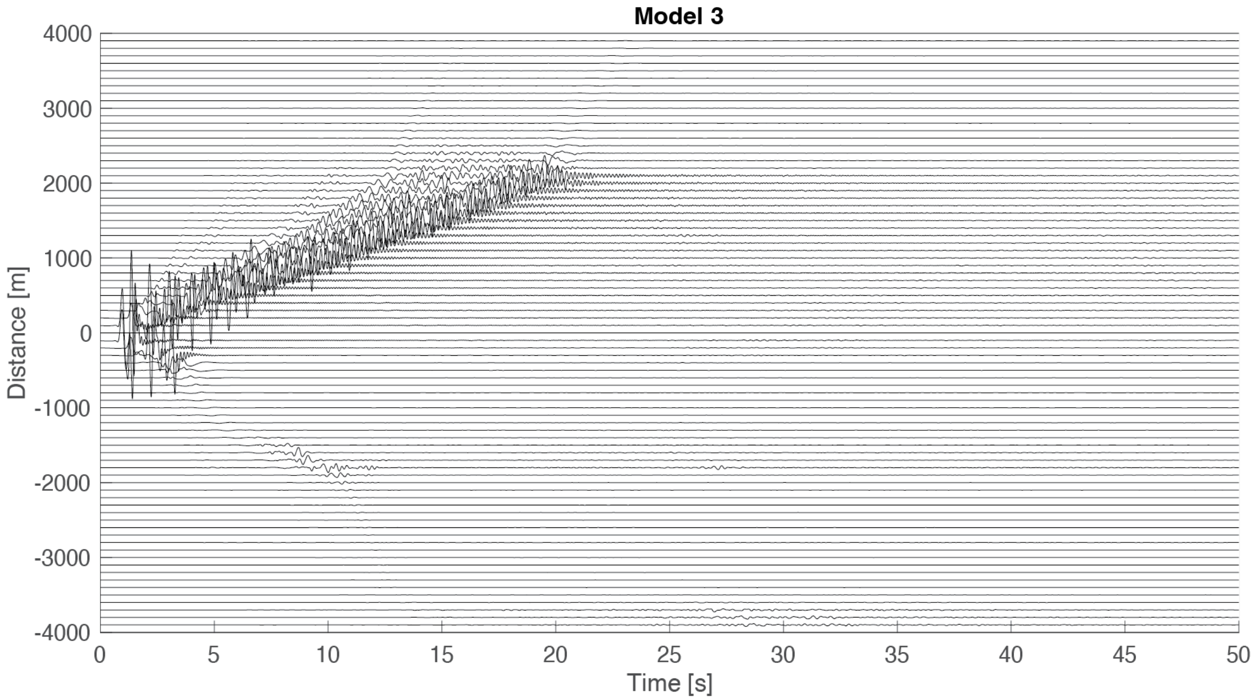

Supplementary Fig S10. Synthetic displacement waveforms for the vertical component of motion for Model 3. Amplitudes are normalized to the peak source and scaled by a factor of 100. The source waveform is not shown as it saturates out of scale.

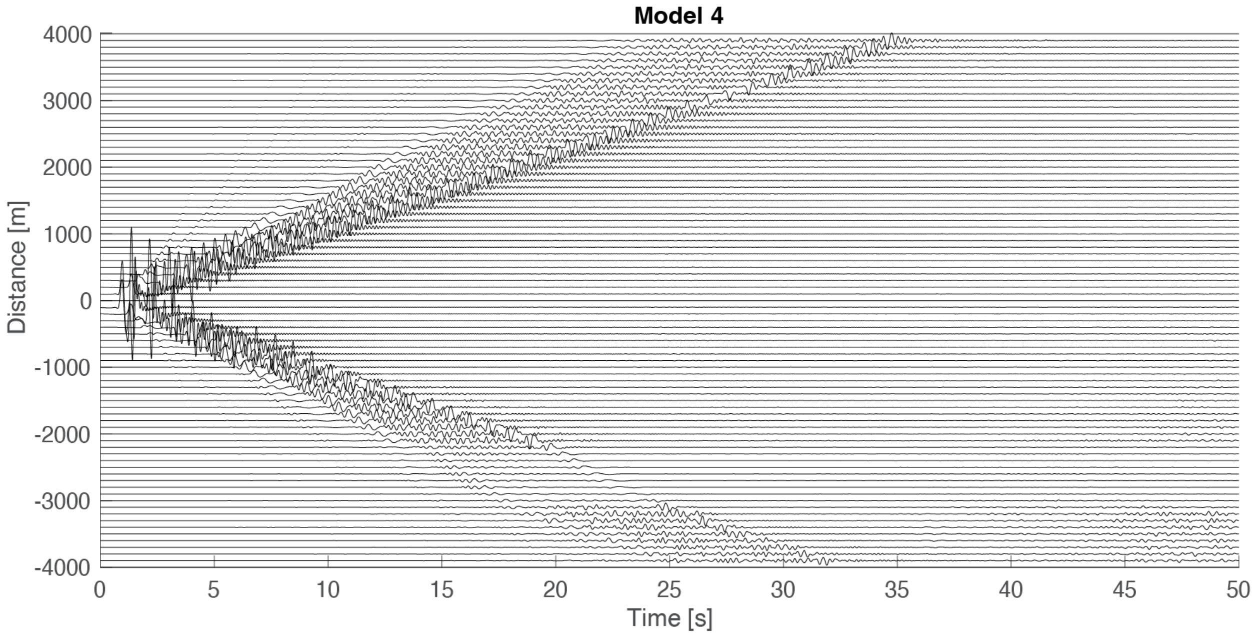

Supplementary Fig S11. Synthetic displacement waveforms for the vertical component of motion for Model 4. Amplitudes are normalized to the peak source and scaled by a factor of 100. The source waveform is not shown as it saturates out of scale.

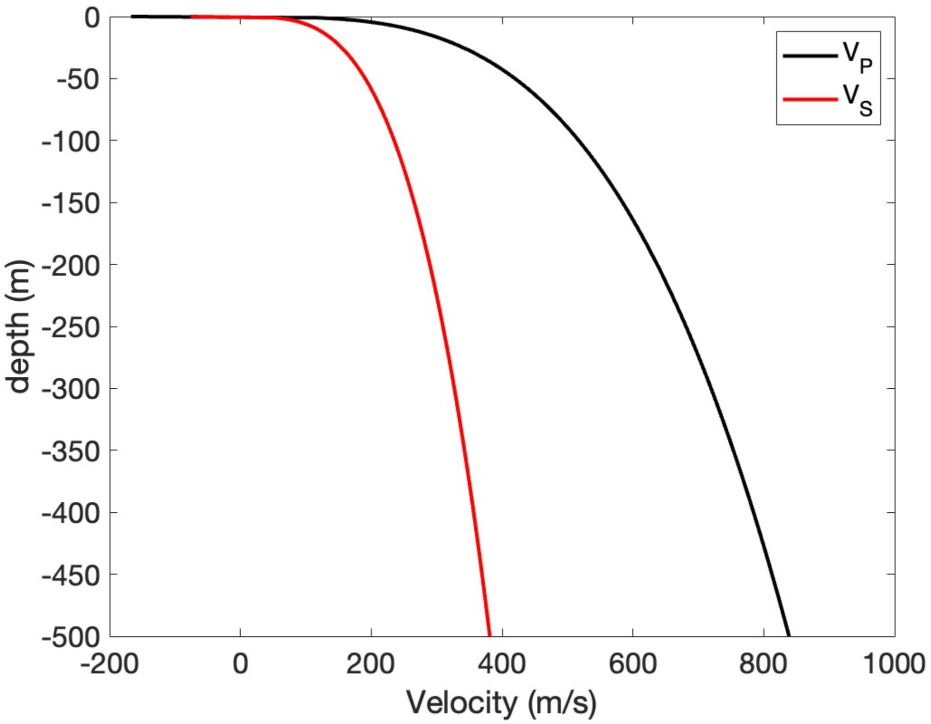

Supplementary Fig S12. Average 1D velocity profiles for all velocity models. Models used for seismic simulations are created by adding velocity anomalies according to our thermal and rock physics model.
